# Vaccination policy strategies before and during the COVID-19 pandemic: an overview: Jacopo Garlasco

**DOI:** 10.1093/eurpub/ckac130.232

**Published:** 2022-10-25

**Authors:** J Garlasco, L Charrier, R Thomas, P Gardois, M Bo, CM Zotti

**Affiliations:** 1 Department of Public Health Sciences, University of Turin, Turin, Italy; 2 Northern Metropolitan Department Direction, Local Health Authority Turin 3, Venaria Reale, Italy; 3 Biblioteca Federata di Medicina F. Rossi, University of Turin, Turin, Italy; 4 Hospital Medical Direction, Local Health Authority Turin 5, Chieri, Italy

## Abstract

**Background:**

The debate on vaccination strategies has been periodically involving researchers, policymakers, and also the population. Interest waves have occurred both after a revival of childhood infectious diseases in 2016-2017, due to low vaccine coverages, and during the recent Coronavirus outbreak. This study aimed at overviewing vaccination strategies (and corresponding vaccine coverages) for childhood vaccinations and SARS-CoV-2.

**Methods:**

Measles was chosen as a childhood vaccination indicator. Policy data were retrieved from health institutions (either European or national/regional) and, for COVID-19, also from press agencies and newspaper websites. Vaccine coverage data were retrieved from the World Bank, World Health Organisation, and UNICEF databases (for childhood vaccines), and from the “Our World in Data” platform for SARS-CoV-2. A qualitative comparison was performed between the two contexts.

**Results:**

Unlike childhood vaccinations, few countries (and only Austria in Europe) imposed generalised COVID-19 mandates, most countries preferring targeted mandates for higher-risk groups. Many countries confirmed their traditional voluntary vaccination approach also for COVID-19, while countries historically relying on compulsory vaccination strategies, such as Slovenia and Hungary, surprisingly opted for voluntary SARS-CoV-2 vaccination, with unsatisfactory results. However, no tangible crude association was generally found between vaccination policies and achieved coverages, although factors such as cultural background, education, and religion appeared to influence the impact of vaccination policies.

**Conclusions:**

The COVID-19 experience has enriched pre-existent vaccination strategy debates by adding interesting elements concerning attitudes toward vaccines in a novel context. Reading the available results in the frame of vaccine hesitancy determinants can help to understand the relationship between policies and actual coverages.

**Key messages:**

- Few countries have imposed generalised mandates for COVID-19, while many preferred targeted obligations for high-risk groups and some countries surprisingly opted for a voluntary approach.
- Accurately considering social and cultural determinants allows understanding the chance of success of vaccination strategies, ahead of fostering the right policymaking approach for each population.

